# Development and optimisation of in vitro sonodynamic therapy for glioblastoma

**DOI:** 10.1038/s41598-023-47562-2

**Published:** 2023-11-18

**Authors:** Andrew Keenlyside, Theodore Marples, Zifan Gao, Hong Hu, Lynden Guy Nicely, Joaquina Nogales, Han Li, Lisa Landgraf, Anna Solth, Andreas Melzer, Kismet Hossain-Ibrahim, Zhihong Huang, Sourav Banerjee, James Joseph

**Affiliations:** 1 Centre for Medical Engineering and Technology (CMET), University of Dundee, Nethergate, Dundee, DD1 4HN UK; 2 Department of Cellular and Systems Medicine, School of Medicine, University of Dundee, Dundee, DD1 9SY UK; 3 Department of Biomedical Engineering, School of Science and Engineering, University of Dundee, Dundee, DD1 4HN UK; 4 Innovation Center Computer Assisted Surgery, Institute at the Faculty of Medicine, Leipzig University, 04103 Leipzig, Germany; 5 Department of Neurosurgery, Ninewells Hospital and Medical School, NHS Tayside, Dundee, DD1 9SY UK

**Keywords:** Cancer, Engineering

## Abstract

Sonodynamic therapy (SDT) is currently on critical path for glioblastoma therapeutics. SDT is a non-invasive approach utilising focused ultrasound to activate photosensitisers like 5-ALA to impede tumour growth. Unfortunately, the molecular mechanisms underlying the therapeutic functions of SDT remain enigmatic. This is primarily due to the lack of intricately optimised instrumentation capable of modulating SDT delivery to glioma cells in vitro. Consequently, very little information is available on the effects of SDT on glioma stem cells which are key drivers of gliomagenesis and recurrence. To address this, the current study has developed and validated an automated in vitro SDT system to allow the application and mapping of focused ultrasound fields under varied exposure conditions and setup configurations. The study optimizes ultrasound frequency, intensity, plate base material, thermal effect, and the integration of live cells. Indeed, in the presence of 5-ALA, focused ultrasound induces apoptotic cell death in primary patient-derived glioma cells with concurrent upregulation of intracellular reactive oxygen species. Intriguingly, primary glioma stem neurospheres also exhibit remarkably reduced 3D growth upon SDT exposure. Taken together, the study reports an in vitro system for SDT applications on tissue culture-based disease models to potentially benchmark the novel approach to the current standard-of-care.

## Introduction

Glioblastoma (GBM) is a highly invasive and refractory grade IV glioma with extremely poor median patient survival of 12–15 months from initial diagnosis^1, 2^. A GBM tumour is hypoxic with microenvironment niches exhibiting considerable genetic instability and neovascularisations leading to highly heterogenous tumour progression^3^. The majority of the tumour comprises of glioma stem cells which exhibit a diverse transcriptome with remarkable reciprocity which grants effective resistance to many traditional and novel treatment options^4, 5^. Furthermore, complete surgical debulking is often impossible as the tumour invades healthy brain by hijacking normal neuronal functions^6^. However, surgical interventions have improved over the past years, especially with the FDA approval of 5-Aminolevulinic Acid (5-ALA) in 2017, a photosensitizer that allows surgeons to delineate normal brain tissue from glioblastoma.

5-ALA is a metabolic intermediate in the heme pathway which is upregulated in GBM^7^. Upon oral intake, exogenous 5-ALA crosses the inflamed blood–brain barrier (BBB) and selectively accumulates in the tumour^8^. GBM-specific upregulation of the heme pathway promotes rapid metabolic breakdown of 5-ALA into the photosensitiser protoporphyrin IX (PpIX)^9^ which exhibits a selective accumulation within the tumour^10^. Since PpIX is fluorescent under 405–633 nm light^9^, it effectively provides intra-operative guidance for tumour resection^11^. Although fluorescent-guided tumour resection has improved patient progression-free survival (time to relapse), this has yet to be shown to increase overall survival^12^, with over 80% of tumour recurrence occurring within 2 mm of even completely resected tumour margins^13^. Hence, multiple studies have suggested exploiting the selective accumulation of photosensitiser and induce death in the residual tumours using photodynamic therapy (PDT)^14^. While PDT could indeed be a potential new therapeutic approach for GBM it would require craniotomy for effective tumour targeting using laser light to activate 5-ALA, thus ruling out repeated regular dosing. Interestingly, in 2014, 5-ALA was reported to be a sonosensitiser which opened up the possibility of non-invasive excitation of the 5-ALA-metabolite PpIX using focused ultrasound^15, 16^. Focused ultrasound has been FDA and EMA-approved and is used for hyperthermic thalamotomy for essential tremor and BBB modulation^17, 18^ When applied together with sonosensitisers, it is referred to as sonodynamic therapy (SDT).

The use of SDT as a cancer therapy procedure is under investigation. However, the mechanism of FUS-mediated photoexcitation of PpIX remains unknown. Several mechanisms may contribute to activation^16^. Mechanisms involving microbubble collapse generating blue channel photoemissions and direct mechanical effects have been discussed^19^. These photoemissions cause the development of primarily mitochondrial singlet reactive oxygen species (ROS)^20^. Subsequent cell cycle arrest and caspase activation cause the selective death of tumour cells, sparing normal neural cells which do not contain elevated PpIX concentrations^21^. Multiple other cytotoxic pathways may be implicated, with different GBM subclones showing variable responses to ROS^22, 23^.

Recently, SDT studies showed positive outcomes from in vivo and clinical settings. Significant improvements to survival and tumour inhibition were seen in SDT-treated glioma rat models utilising a 20-min sonication (1.06 MHz, 5.5 W/cm^2^) with + 2.5 °C of sub-ablative tumour hyperthermia. In this study, 5-ALA was injected 6 h prior to sonication at a dose of 60 mg/kg into C6 rat allografted glioma cells^24^. The rat studies also observed benefit when using pulsed FUS treatments (86 ms, 8.6% duty cycle)^25^. These successes led to the instigation of the first phase-0 human trials (clinical trial ID: NCT04559685). Initial results indicate this treatment is safe with its efficacy to be evaluated in the coming years. Future optimisation studies in SDT are expected to focus on low-intensity FUS (< 25 W/cm^2^ in humans, < 10 W/cm^2^ in vitro). Although SDT has been rapidly taken to human trials, the exact molecular mechanisms leading to this combined effect of FUS and sonosensitisers in malignant glioma remains unclear. No optimum in vitro setup and related cell sampling techniques for in vitro SDT has been systematically optimised. Additionally, the effect of SDT on primary patient-derived glioma stem cells in vitro has never been studied. This paper optimises various systems parameters such as the plate material and thickness, acoustic intensity, thermal effects, ultrasound frequency, and methods to reduce pressure distortion due to the formation of acoustic standing waves and eventually reports systematic cell-based studies to validate the experimental SDT system. Taken together, the current study reports a comprehensive system for in vitro SDT and establishes the first systematic testing and cell death investigations in state-of-the-art primary patient derived glioma cell and stem cell models.

## Methods and materials

### The SDT experimental setup

**Figure 1:**
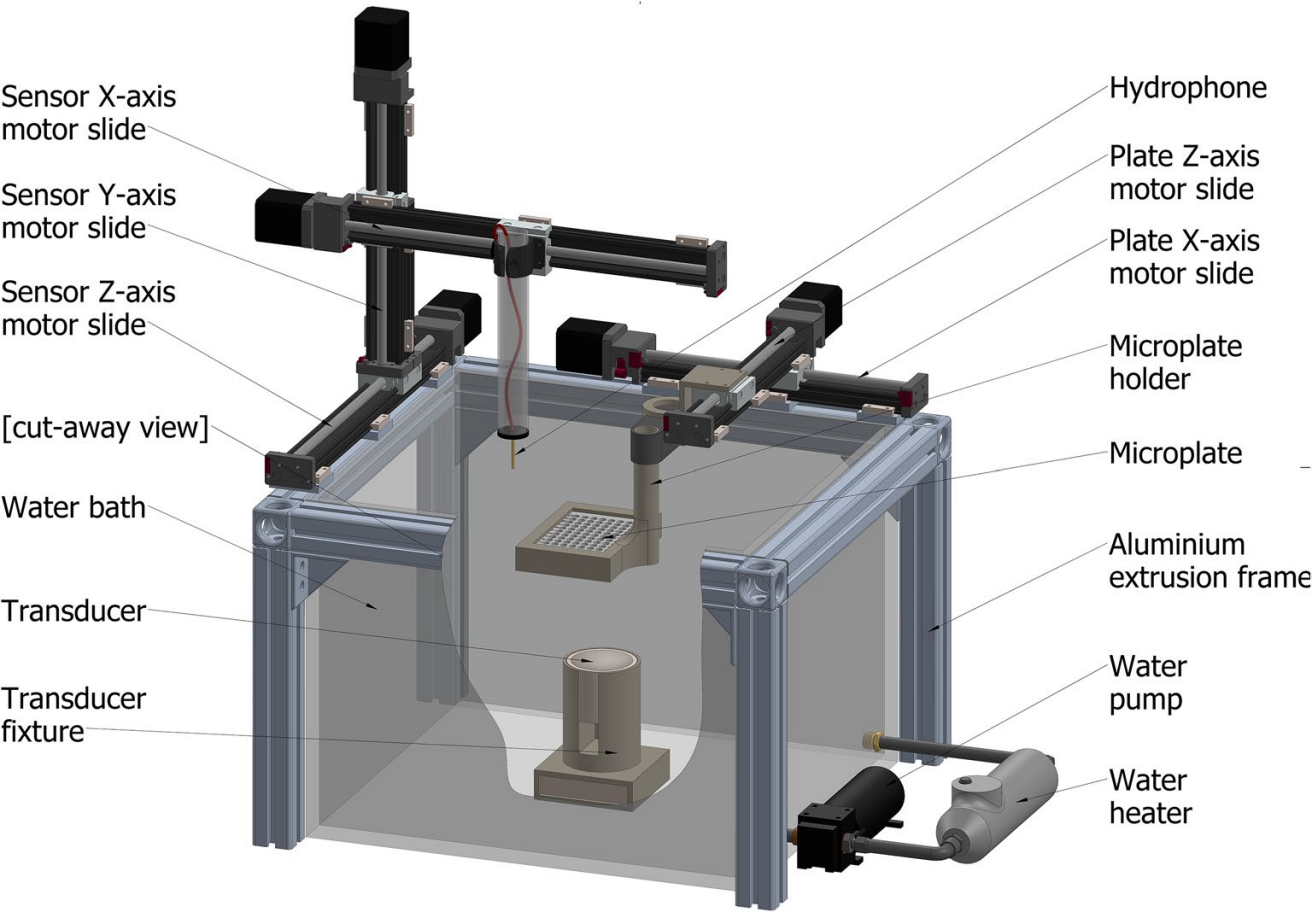
Illustration of the in vitro experimental setup: water bath, needle hydrophone, plate holder and 96-well plate, transducer and fixture, sensor motor slides for the X, Y, and Z axis, and water heater and pump. See also Supplementary Table S1.

Figure 1 illustrates the experimental setup. Briefly, the experimental SDT system comprised of an acrylic water bath (423 mm × 486 mm × 302 mm) constructed with an open top and an external water loop with an in-line water heater (T08200 ETH 300W, Hydor) and pump (LET 775, 12 V, 65PSI, Lei Te Co.) to circulate temperature-controlled water. The design featured two motorised scanning platforms, one with three degrees-of-freedom (DOF), and another with two-DOF. The former scanning platform was used to perform US field mapping using a needle hydrophone (NH) (D = 1 mm, Precision Acoustics), mounted onto a custom 3D printed bracket. The field mapping platform had motion across the X, Y and Z axes. NH output signals were acquired using a high-resolution oscilloscope (PicoScope® 4224, Pico Technology) and relayed to a PC for data logging using a custom MATLAB programme. Well plates (*Standard clear 96-well plate with 1 mm polystyrene-base,* ThermoFisher Scientific, product ID: 167008 or a ND *Ultrasound-translucent 96-well plate:* (µClear®, 190 µm film-based), Greiner Bio-One, product ID: 655097) were mounted using custom, 3D printed, brackets to the latter stage and had motorised range in the X and Y axes. This bracket could be manually adjusted along the Z axis to position the wells in the focal range of the ultrasound transducer. Both stages were translated using motorised linear slides (Motorized XSlide™, XN10 Series, Lead Screw, Velmex Inc.) driven by stepper motor controllers (VXM™-2 Motor Controller, Velmex Inc.) and driven by a custom MATLAB programme. A thermal camera (thermoIMAGER TIM 160, Micro-Epsilon UK Ltd.) was mounted, fixed relative to the water bath, and manually focussed on the target. Two ultrasound transducers of differing frequencies (666 kHz, Precision Acoustics UK, and 1193 kHz, homemade) were used. They were interchangeable prior to any given test and were mounted in the water bath underneath the stages. The ultrasound transducers were driven using a signal generator (AFG3101, Single Channel, Arbitrary/Function Generator, Tektronix UK Ltd.). PVA gel was synthesised for a specific testing process outlined below using polyvinyl alcohol (Poly(vinyl alcohol), 99.3–100.0% hydrolysed, M.W. approx. 146,000–186,000, ACROS Organics™, Fisher Scientific UK).

### Needle hydrophone field mapping

The method for mapping a 2D area of the ultrasound field was based on sampling the pressure fields using a hydrophone moved at incremental steps along a given axis (X) until the width of the chosen area had been covered. The hydrophone was jogged by one increment (0.1 mm) in a chosen perpendicular axis (Y), before repeating the incremental sampling across the width in the opposite direction, X. This process was repeated until the desired height was covered, generating a grid of data in the XY plane with a resolution equal to the increment length. This process was executed using a custom programme written in MATLAB. By assigning a colour gradient to the spectrum of pressure values generated, a visual representation of the ultrasound field in the XY-plane was produced. This mapping method was applied to the following experiments to produce field maps in all three cardinal planes (with the exception of the PVA experiment). Four distinct exposure setups were devised to examine the effects of altered system parameters:

*No well plate:* The well plate was removed from the testing setup to allow the open field to be mapped within the water bath, with the water level well above the focal point of the US field. The focal point was identified by manually jogging the hydrophone until the peak sound pressure was located. This also served as a reference point for the centre of the mapped area (supplementary Fig. S1A).

*In well (water level):* The well plate and thermal camera were included in the testing setup and the stage was adjusted to place the well plate at the level of the water surface and with the focal point of the ultrasound within the target well. The thermal camera was focussed on the target well which was filled with 400 μL water. Surrounding wells contained 100 µL of phosphate buffered solution (PBS) (supplementary Fig. S1B).

*In well (submerged):* The well plate was included in the testing setup and the stage was adjusted to place the well plate 60 mm below the water surface and with the focal point of the ultrasound within the target well (supplementary Fig. S1C).

*In well (PVA—Submerged):* PVA negative casts of the wells were made to study the effects of different ultrasound reflection coefficients within the wells. The casting process is described later. To map the field within the PVA material, a pinhole was created in the centre of one well’s cast, allowing the tip of the hydrophone to be inserted into the well. In this method the movement of the probe was constrained to the Y axis. The results were plotted as a line graph of pressure readings against the height above the floor of the well.

### 2D cell culture reagents and assays

Primary patient-derived glioblastoma cell GBM22 was acquired from the Mayo Clinic, USA and cultured as reported previously^26, 27^. 5-Aminolevulinic acid hydrochloride > 98% (5-ALA) (Sigma Aldrich, St. Louis, Missouri, United States) was used for cell dosing alongside standard GBM media. GBM22 cells were plated with 50 μL of standard GBM media in standard clear tissue-culture treated 96-well plates described above or 18-well chambered coverslips (Ibidi, Munich, Germany). 5-ALA was diluted in GBM media to 2 mM and added to cells at a final concentration of 1 mM 2 h prior to sonication. Non-ALA treated controls were supplemented with 50 μL of GBM media alone. For the assessment of reactive oxygen species, CellROX Green (#C10444; ThermoFisher Scientific, Waltham, MA, United States) was added to both 5-ALA stock solutions and GBM media intended for non-ALA conditions as per manufacturer’s instructions. For the assessment of apoptosis, Annexin V FITC (Abcam, Cambridge, UK) was used. Using suction, all media was removed from the wells within 15 min of the completion of sonication. The wells were washed with 50 μL buffer solution, which was then removed before the addition of 25 μL of buffer solution containing Annexin V-FITC diluted as par manufacturer’s instructions. The cells were incubated for 90 min prior to microscopy. Annexin V and CellROX agents were never used together. Bright field and fluorescent imaging were taken using a Thermo Scientific EVOS imaging system.

### 3D glioma stem neurosphere culture and treatments

GBM120 3D glioma stem neurosphere line were acquired from the Mayo Clinic, USA and cultured as reported previously^27^. Neurosphere cultures, for either formation or formed assay, were plated with poly-D-lysine (PDL) (ThermoFisher Scientific, Waltham, Massachusetts, United States) to ensure adherence to the base of the well of the 96-well plate. Cultures were then dosed using the above 5-ALA method and treated for 30 s cumulative sonication at standard parameters. No fluorescent reagents/dyes were used in these cultures. Bright field images of the neurospheres were taken at day 0 (pre-treatment) and day 1 onwards every 3–4 days until 21 days using a Thermo Scientific EVOS imaging system. Formation assays were treated 1 day after plating whilst formed assays were treated after 21 days of culture development and monitored for a further 21 days.

All primary glioma cells were derived from the resected tumours with approval from the Mayo Clinic Institutional Review Board (IRB) and written consent from participating, informed, adult patients. The cells were provided by Dr. Jann Sarkaria, Mayo Clinic, USA through a material transfer agreement and all methods were carried out as per guidelines of the IRB.

### Sonication of cells

Sonication of cell lines followed a standardised set of US exposure parameters: 0.4 W/cm^2^, 10% duty cycle, 90 ms pulse length. These were continued until either 30 s or 60 s of cumulative sonication had been completed (5 or 10 min of treatment respectively) unless otherwise stated in the figure legends. The water bath was maintained at 37 °C. These tests were conducted in TC treated polystyrene plates using the *In well (water level)* setup and 666 kHz transducer. The overall energy applied to cells at the centre of each well was estimated to be 1.5 J (for 30 s cumulative sonication).

### Antibodies and immunoblotting

1.5 h post treatment, the cells were lysed in 1 × western blot loading buffer (5x: Tris pH 6.8; SDS 10%; glycerol 30%; bromophenol blue 0.02%; beta-mercaptoethanol 5%). Buffer components were purchased from Sigma Millipore. Immunoblotting was carried out as discussed previously^28, 29^ using the following antibodies: total AKT (Cell Signaling #9272), phospho Thr308 AKT (Cell Signaling #2965), phospho Ser473 AKT (Cell Signaling #9018), phospho Thr202/Tyr204 p44/42 ERK1/2 (Cell Signaling #4376), and GAPDH (Cell Signaling #5174).

### System calibration

The SDT system was calibrated to ensure accurate FUS dose estimation and concordance to pressure field simulations. The system can be calibrated manually or automatically along X, Y, or Z axis, this method allowed accelerated testing protocols when the transducer position remained constant (Supplementary Fig. S2). There was negligible difference in peak pressure between calibration methods (121 kPa with continuous wave 0.4 W/cm^2^). The diameter of the focus point may be altered by transducer frequency and curvature of the transducer surface. When a 666 kHz transducer is utilised, the focus point diameter was measured to be 4.5 mm, slightly narrower than the inner diameter of a typical 96-well plate well. The effect on adjacent wells is minimal (Supplementary Figs. S3).

### Simulation and data analysis

US field simulation models based on finite element analysis were developed in COMSOL Multiphysics v5.4 (ANSYS Inc.). The transducer, tank environment, and one microplate well were modelled using their realistic properties and assigned with custom material properties sourced from the data sheets of each product (Pz54 and Pz26 piezoelectric transducer material, HD polystyrene microplate, and acrylic tank). The model was meshed to a maximum element size of 0.6 mm for each simulation. The “pressure acoustics, frequency domain” model was used to generate the acoustic physics of the transducer. Analysis of NH field mapping data and the production of relevant figures was completed in MATLAB (MathWorks, Massachusetts, USA). Image J (National Institute of Health, Maryland, USA) was used to assess neurosphere diameter. PRISM—GraphPad (California, USA) was used to generate data characteristics and apply statistical analyses.

## Results

### The use of pressure field simulations

**Figure 2:**
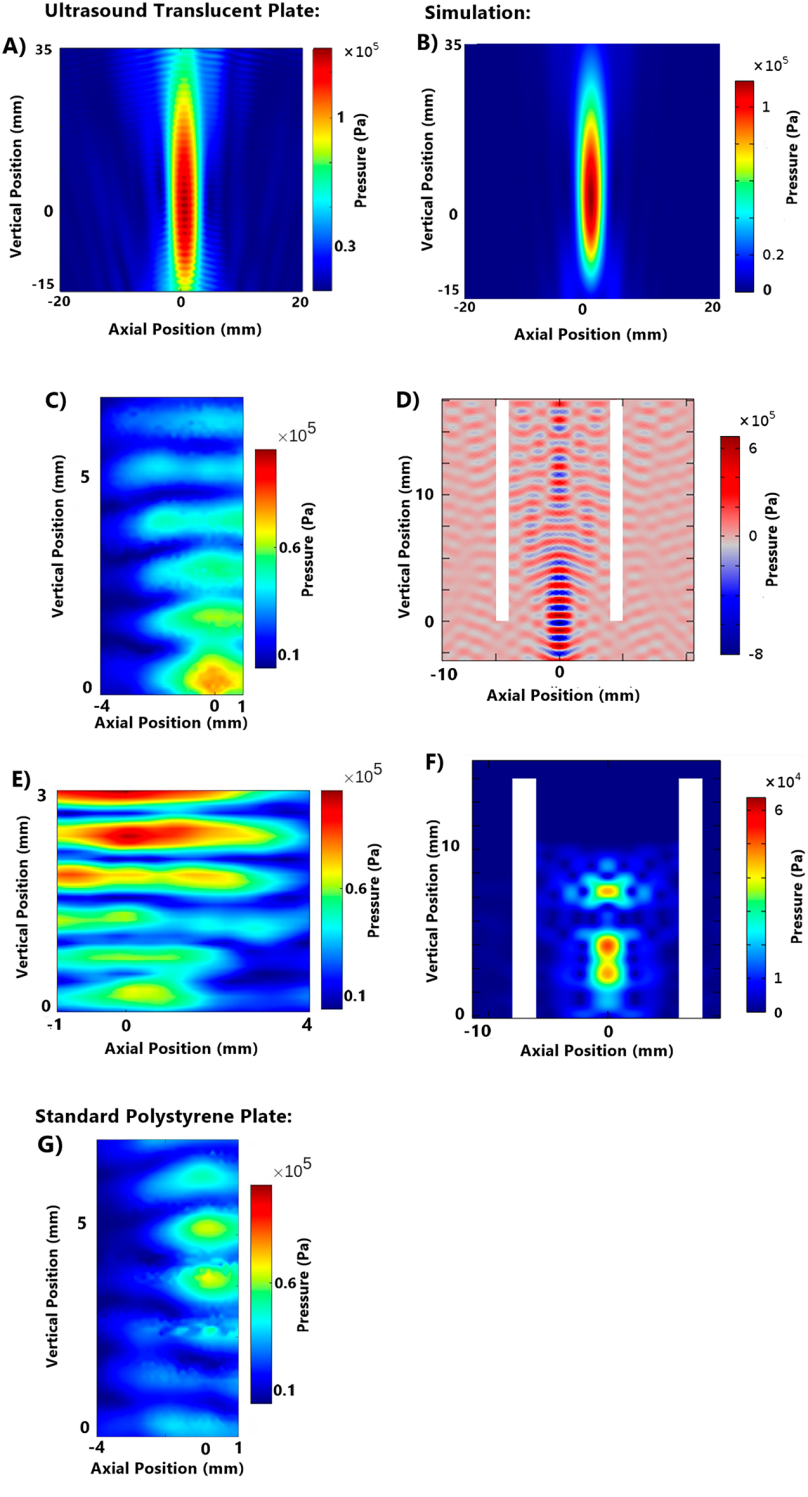
Comparison between ultrasound translucent plate NH mapping and simulation for no well plate, in-well (submerged), and in-well (water level) setups and standard polystyrene plate in-well (submerged setup). 0.4 W/cm^2^ continuous wave. Simulations completed in COMSOL. (**A**) NH field mapping: no well plate. (**B**) Field Simulation: no well plate. (**C**) NH field mapping: in-well (submerged) in an ultrasound translucent plate (190 µm film-base). (**D**) Field Simulation: in-well (submerged). (**E**) NH field mapping: in-well (water level). (F) Field simulation: in-well (water level). (**G**) NH field mapping: in-well (submerged) in a standard polystyrene plate (1 mm base thickness).

Pressure field simulations were initially performed to optimise the experimental setups and estimate the US exposure levels on the cells and assess the impact of materials in an in vitro setup. Field simulations were also used where field mapping using a hydrophone was not feasible or would alter the pressure field. For field simulations to be a useful tool they must be concordant with the NH field mapping approach to provide comparable results. Simulations and field mapping of three different test setups were undertaken to measure the concordance of the results (Fig. 2). The setups tested were, (1) transducer field without a plate or any materials in place, (2) in-well testing of a submerged plate 60 mm below the water bath surface, and (3) in-well testing of a plate resting on the water level with 400 µL of water in each well.

Simulation results showed good concordance to the in vitro field mapping (Fig. 2). In-well (submerged) and in-well (water level) NH field mappings gave comparatively limited field mapping capabilities within the wells, when compared to simulation, due to the risk of contact damage to the needle hydrophone. In both NH mapping and simulation, in-well (submerged) study showed a peak pressure at the base of the well which then weakens. Simulations also showed an increase in pressure levels further above the level visible in NH mapping, highlighting the expanded map that simulations provide. In-well (water level) simulations assumed media filled adjacent wells and showed consistent properties of pressure dampening across the lateral walls of the well plate. This can be contrasted against NH field mapping which illustrates the effect, until 1 mm above the base of the well. This change is due to the fluid level in the adjacent well containing 100 μL compared to the 400 μL in the target well required for NH mapping.

### Plate base material and thickness

**Figure 3:**
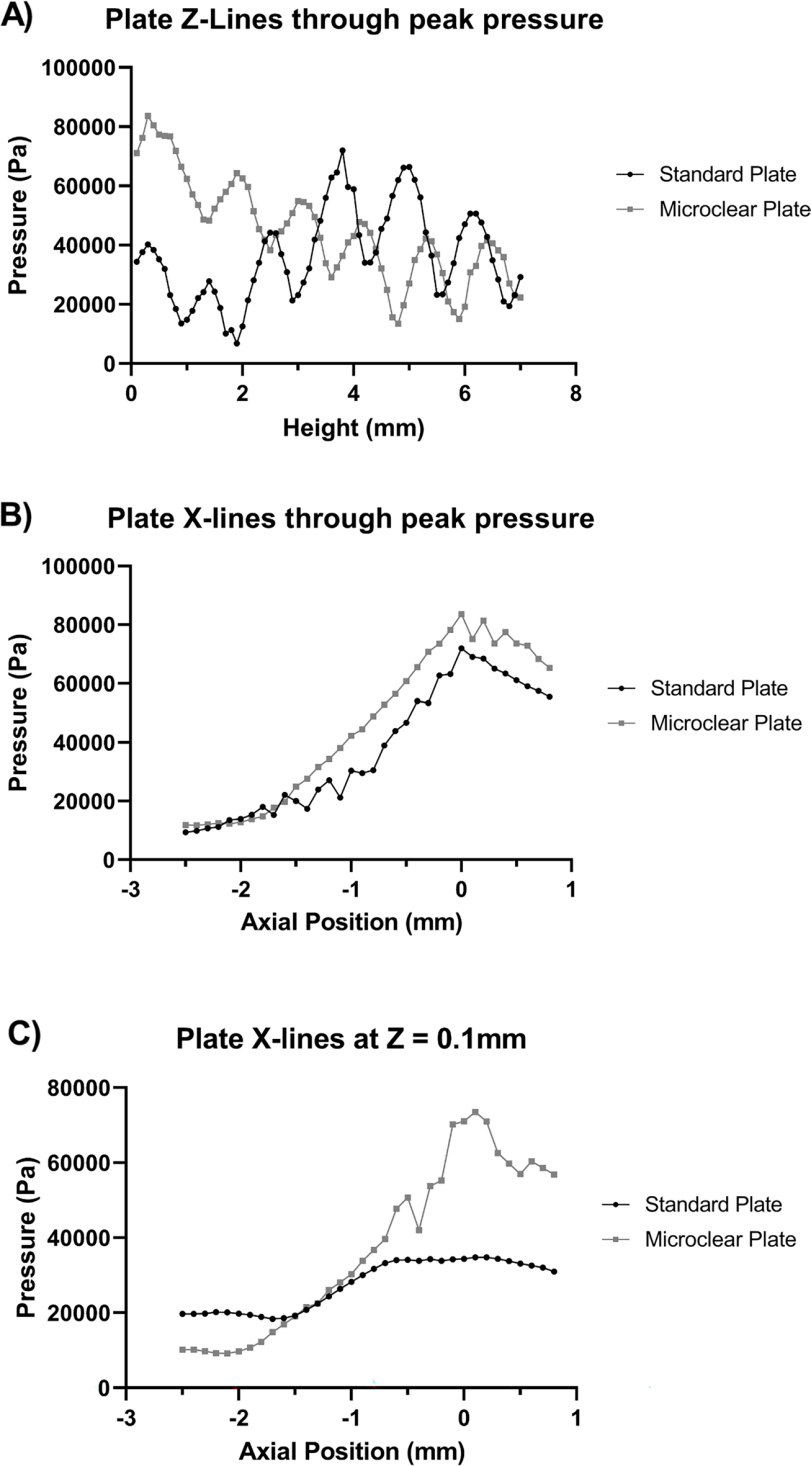
Single axis pressure profiles to compare standard polystyrene and ultrasound translucent (MicroClear) plates. (**A**) Z-axis profiles intercepting the peak pressure point. (**B**) X-axis profiles intercepting the peak pressure point. (**C**) X-axis profiles at Z = 0.1 mm, the height of the cells in the well.

Standard tissue culture treated sterile plates made from clear moulded polystyrene were used. The base of the well had an average thickness of 1 mm. Acoustic boundaries formed due to the finite thickness of the well base resulted in the distortion of the pressure field within the well. Therefore, well plates with thinner bases were considered as a potential alternative for performing in vitro focused ultrasound studies. The in-well (submerged) ultrasound fields of both standard polystyrene 96-well plates and ultrasound translucent (microclear) plates, were compared to determine the impact of base polystyrene on ultrasound field distortion within a well. Continuous wave FUS at 5.5 W/cm^2^ was used for the studies. NH pressure field mappings performed on both plates under the described conditions showed clear differences in US field distribution within the standard and microclear well plates (Fig. 2). Single axis field profiles were also measured (Fig. 3) along the z-axis through the centre of the well x-axis profiles at the level of each plate’s peak and 0.1 mm above the base of the well plate, which formed the location of the cultured cell. The studies quantified the difference in height above the bottom of the well that each peak occurred at and illustrated the difference in pressure variability over the height of the well. The microclear plate gave a coefficient of variation (CoV) of 38.1% along the vertical axis; lower than the standard plate which offered CoV of 42.9%. A lower coefficient of variation in this experiment indicates reduced variation in the pressure field and demonstrates the impact of base thickness on acoustic field distribution.

Investigations were also performed to assess the field distributions in the region immediately above the base of the well, where the cell cultures are fixed. Increased acoustic translucency was observed in the microclear plates (Fig. 3). Further, the line profiles were found to be similar in shape and magnitude to the x-line at peak intensity, as illustrated in Fig. 3. This is desirable for precision dosing since doses are calculated from peak intensity values. The reduced acoustic translucency of the standard plate showed flatter field distribution across the cell lines and would therefore provide uniform US exposures across the width of the well. The CoVs of acoustic field distribution at the well base of the standard plate was observed to be 23.7%, compared to 59.2% with the microclear plate. However, the CoV of field distribution in microclear plates were reduced to 18.9% when the radius was limited to 0.8 mm which closely matches the US beam profile.

### Acoustic intensity, pressure, and thermal effects

Acoustic intensity variations could also impact the distribution of acoustic pressure distribution due to non-linearities in absorption and scattering during a FUS exposure. Further, changes to the distribution of the ultrasound field at varying acoustic intensities could impact the ultrasound dose delivered to cells across experiments. However, at low acoustic intensities, such as those intended for the SDT proposed in this paper, the impact of ultrasound field distribution and dosage at varying acoustic intensities is not well established^30^. Therefore, systematic studies to assess the pressure field distributions at varying acoustic intensities were also performed.

**Figure 4:**
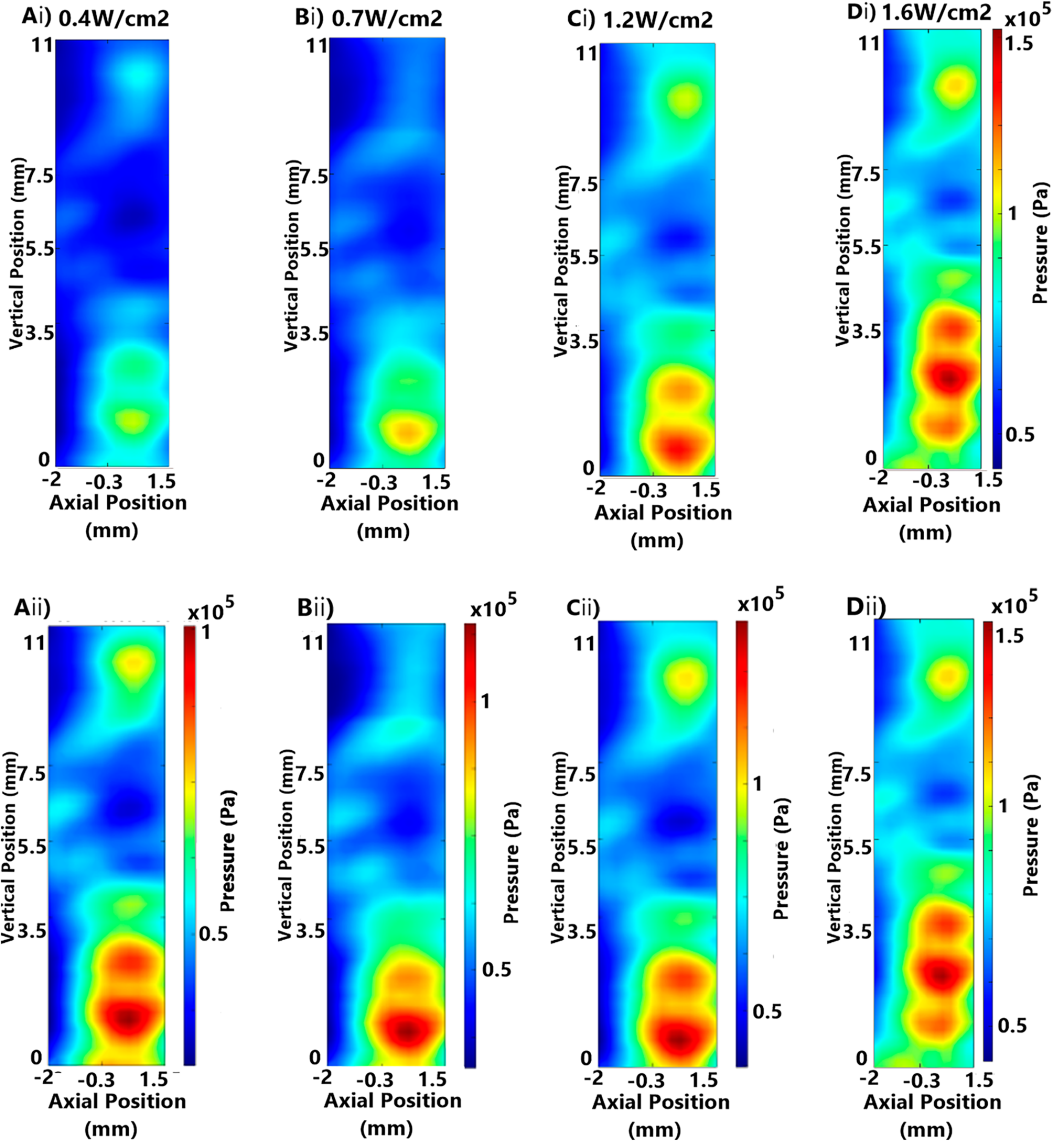
Comparison of in-well (submerged) pressure field mappings at increasing emitted intensity, emitted generator amplitude, and target peak pressure of continuous wave focused ultrasound, with and without standardised scale bars (i and ii subfigures, respectively): (**Ai** and **Aii**) 5.4 W/cm^2^ (2.5 V, 0.402 MPa), (**Bi** and **Bii**) 7.0 W/cm^2^ (2.75 V, 0.458 MPa), (**Ci** and **Cii**) 9.9 W/cm^2^ (3.25 V, 0.545 MPa), (**Di** and **Dii**) 12.2 W/cm^2^ (3.5 V, 0.603 MPa).

In-well submerged FUS field mappings were carried out at varying acoustic intensities of 0.4–1.6 W/cm^2^ with the water level set to 60 mm above the 75 mm focus point of a 666 kHz transducer. The mapped peak pressure at each intensity shows a progressive increase in the peak pressure within the well (100–200 kPa) (Fig. 4Ai–Di). Comparison of non-standardised-scale pressure maps (Fig. 4Aii–Dii) showed a consistent pressure field with increasing intensity, demonstrating that these intensities are sufficiently low to avoid disruption of the field. Therefore, the target pressure and cumulative sonication dose can be varied between wells within a pre-programmed experiment for a rapid testing of parameter configurations.

Further, we also investigated the occurrence of thermal effects due to FUS exposure. High frequency focused ultrasound can carry a significant thermal dose dependant on acoustic intensity and may cause target cell hyperthermia. Regardless of low-intensity ultrasound parameters utilised (< 15 W/cm^2^, < 15% duty cycle), no thermal dose was detectable at the end of a 5-min sonication. The difference between the target well and average temperature of the 8 surrounding wells did not exceed 0.3 °C. Further information is available in the supporting information—*thermal effect* (Supplementary Fig. S4).

### Standing waves and vertical interference

In our system, ultrasound waves meeting the fluid-air boundary at the water’s surface reflect downwards and may interfere with ascending waves to produce standing waves (a phenomenon that is well described in the literature). The degree of ultrasound interference, and thus potential for standing waves, is dependent on the transducer’s curvature and the distance from the focus point to the fluid-air boundary, as the intensity of reflected waves will reduce in line with the inverse square law. Focused ultrasound transducers typically utilise a concave surface to allow the waves to focus to a point; as such a degree of lateral dispersion may be seen beyond the focus point. Therefore, standing waves may be mitigated in submerged experimental setups.

Comparison of the degree of ultrasound field distortion between in-well (water level) and in-well (submerged) setups was undertaken (Fig. 2). The distance between pressure field oscillations increased from 0.5 to 1.2 mm when the plate was submerged (666 kHz transducer), indicating a more consistent pressure field. The diameter of the lowest peak node, indicating the area of cells to which peak pressures have been applied, remained constant however, the in-well (submerged) setups showed a greater intensity at the base of the well while the in-well (water level) setups showed a lower, but more consistent central cell exposure (0.3 MPa compared to 0.25 MPa). The point of peak intensity was raised in the in-well (water level) setup from the base of the well by 2.5 mm.

Further, we also investigated the effect of acoustic boundaries across the wells where multi-well microplates showed varying resonant properties; adjacent wells containing fluid are relatively hypo-resonant, and the air-filled inter-well spaces are relatively hyper-resonant. Observable patterns were produced in the ultrasound field within the target well. Viewed from above, relative ultrasound dampening at 90° increments was seen, in line with fluid-filled wells, and relative ultrasound reflection offset at 45° intervals, in line with the empty inter-well’s spaces. This was also observed in the vertical axis where the height of the fluid level varied between the wells. In NH field mapping, when the target well is filled with the required 400 μl of fluid, and adjacent wells contain just 100 μl of fluid, as shown in Fig. 2, there is a noticeable region of reduced pressure, roughly rectangular in shape, extending inwards from the outside edge of the well by approximately 2 mm, and upwards by approximately 1 mm, corresponding to the height of the fluid in the adjacent well and the boundary between hypo-resonant fluid and hyper-resonant air space. This effect is referred to herein as ‘lateral dampening’.

**Figure 5:**
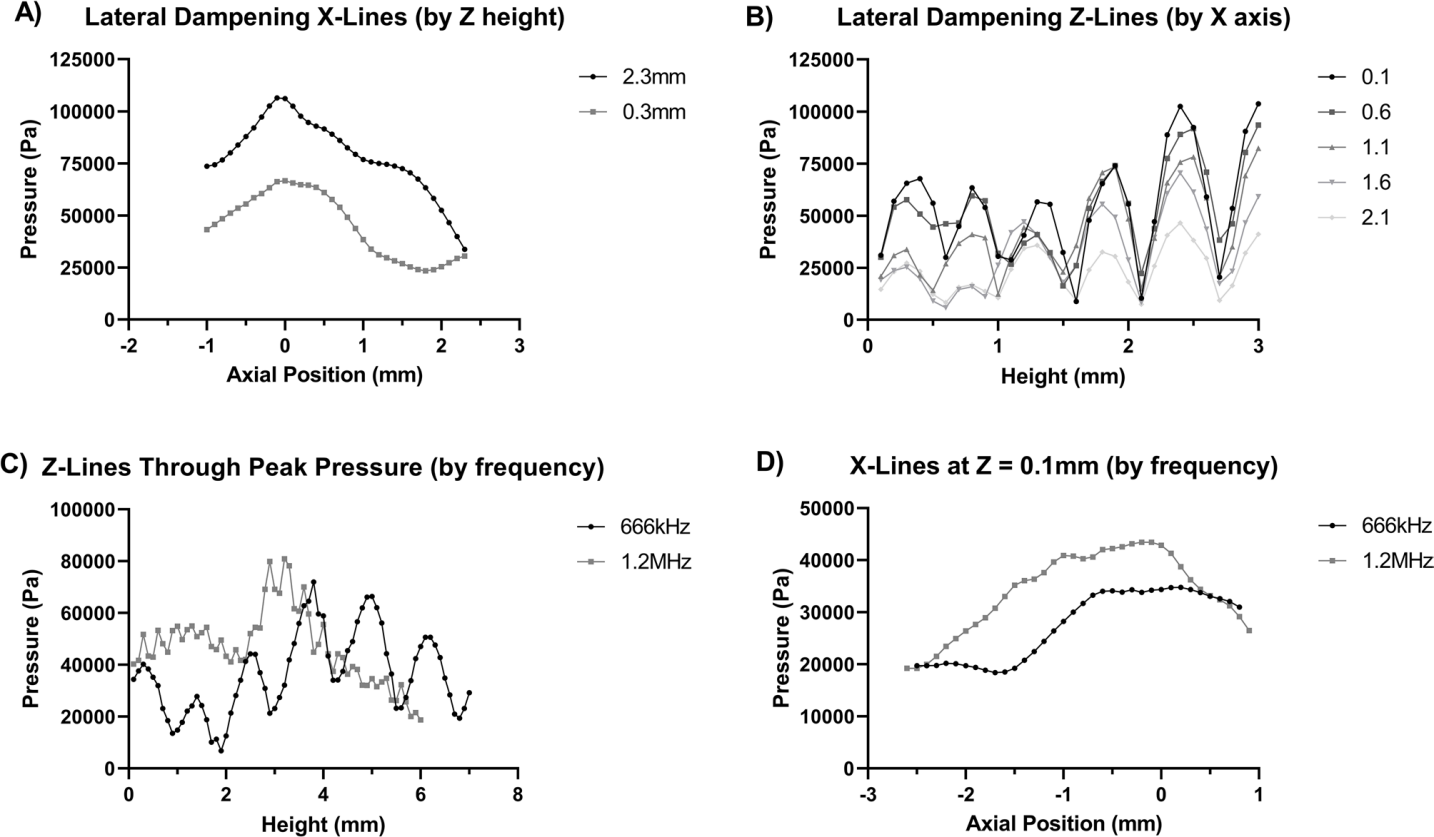
X and Z Pressure profile comparisons illustrating the effects of altered frequency and adjacent structures on pressure distribution. (**A**) X-axis pressure profiles comparing lateral dampening from below and above the water level of adjacent wells, 0.3 mm and 2.3 mm high, respectively, in an in-well (water level) setup. (**B**) Z-axis pressure profiles at 0.1, 0.6, 1.1, 1.6, and 2.1 mm from well central axis for an in-well (water level) setup. (**C**) Comparisons of 666 kHz and 1.2 MHz pressure profiles for 0.4 W/cm^2^ continuous wave focused ultrasound in an in-well (submerged) setup for Z-axis profiles intercepting the peak pressure point. (**D**) Comparisons of 666 kHz and 1.2 MHz pressure profiles for 0.4 W/cm^2^ continuous wave focused ultrasound in an in-well (submerged) setup for X-axis lines at Z = 0.1 mm, the height of the cells in the well.

The lateral dampening effect was also assessed using the two axial profiles taken from the ultrasound field within a well at 0.3 mm and 2.3 mm above the base. As shown in Fig. 5A, lateral dampening was observed with 100 μL of fluid in the adjacent wells. The relative width of the *peaks* of each wave were comparable (taken to be the region of the curve greater than the mean), however, the curve taken at a height of 0.3 mm rapidly attenuated beyond a radius of 0.9 mm. That was not seen in the curve at 2.3 mm height where the field is sustained above − 1 standard deviation until a radius of 1.9 mm. Further, the attenuation of the field was demonstrated using serial z-lines taken at regular intervals from the central axis (Fig. 5B). As expected, above a height of 1.6 mm in the well, the pressure diminished relatively evenly with an increase in the radius (axial distance). As the plot approaches the base of the well (decreasing height), the two innermost radii maintained a regular field pattern. In contrast, the plots at the two outermost radii (and to a lesser extent at a radius of 1.1 mm) were flattened, demonstrating dampening at lower and more lateral points in the well.

It is therefore prudent to be restrictive when sampling: beyond a radius of ~ 0.9 mm, cells do not receive a comparable dose to those within that boundary. The validity of this method is corroborated by the analysis of the microclear plate x-lines, see *‘plate base material and thickness,’* above. Additional measures to mitigate standing waves, including PVA inserts, are discussed in the supporting information (Supplementary Fig. S5).

### Frequency

**Figure 6:**
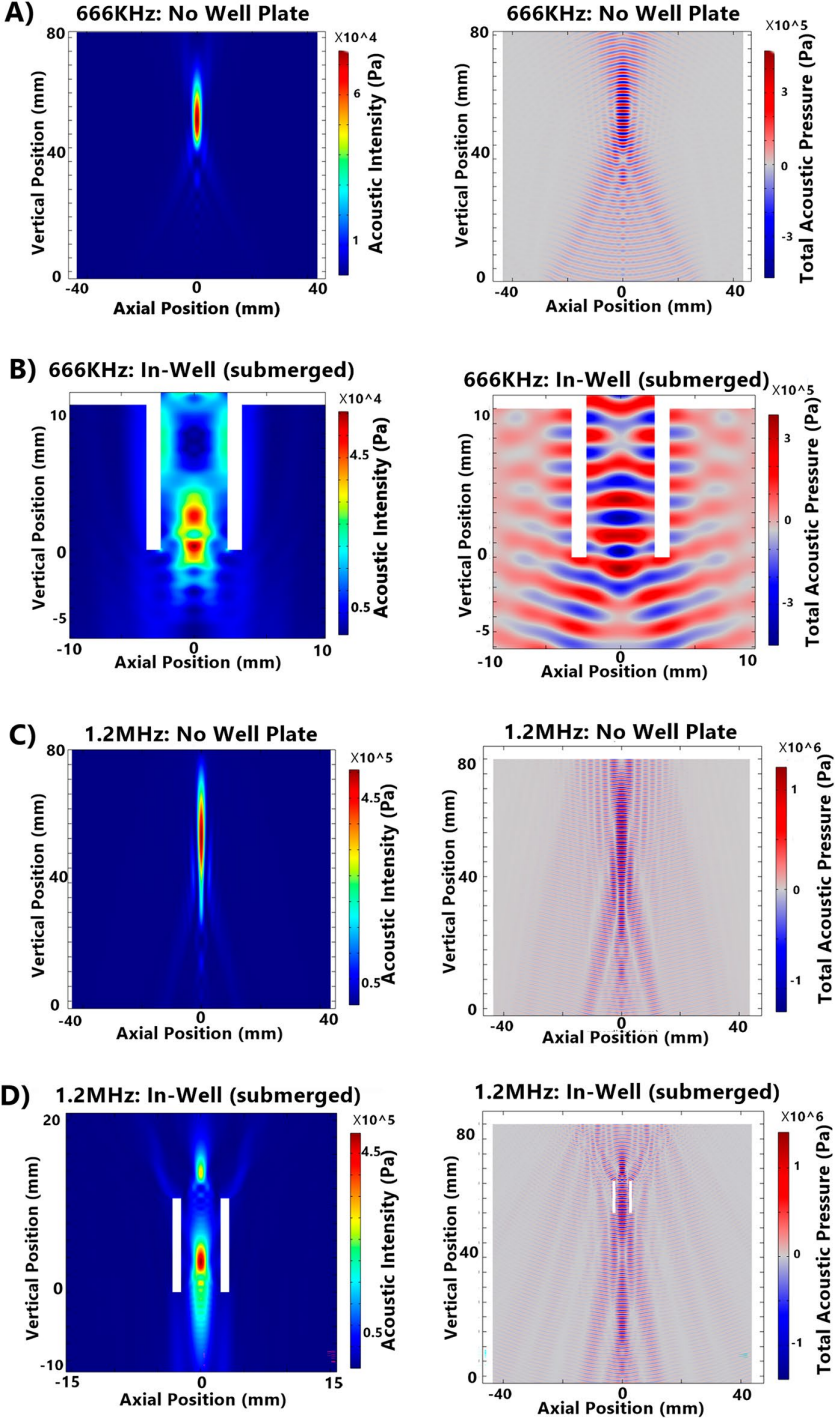
COMSOL Simulations of in-well (submerged) and no well plate setups for 666 kHz and 1.2 MHz transducers. Acoustic intensity and total acoustic pressure are shown for each condition: (**A**) 666 kHz No Well Plate Setup, (**B**) 666 kHz In-Well (Submerged) Setup, (**C**) 1.2 MHz No Well Plate Setup, (**D**) 1.2 MHz In-Well (Submerged) Setup.

Frequency impacts several factors in in-vitro SDT setups. With higher frequency transducers, the focus distance is extended, and the field profile is narrower (Fig. 6A,C). Consequently, the reflected FUS from the fluid-air boundary above ‘doses’ the target well with a higher intensity at higher frequencies (assuming an equivalent submersion depth). The acoustic pressure simulation in Fig. 6 confirmed the presence of the reflected waves, as clearly illustrated in the 1.2 MHz open field image. Changes to frequency would alter the severity of standing wave reflections during the in-well testing with the cells at the water level. 666 kHz and 1200 kHz transducers were compared in 2 settings via the use of pressure field simulations (Fig. 6). For ‘no well plate’ and in-well (submerged) setups, the lower frequency transducer showed a reduction in the frequency of pressure field oscillation and improved consistency for the estimation of FUS dose. In addition to this, the FUS field is broader for the 666 kHz transducer compared to that of the 1.2 MHz transducer. The benefit of a broader field is that the usable cell sample area is expanded owing to a more consistent intensity across the width of the well as described before.

Figure 5C,D shows comparisons of 666 kHz and 1.2 MHz axial pressure profiles for the central Z-axis and X-axis 0.1 mm above the base of the well plate, respectively. While both frequencies demonstrated oscillations throughout Fig. 5C, the amplitude of 1.2 MHz field was observed to be lower. It should be noted that the magnitudes of these graphs are not comparable since the applied voltages across the transducers were different. By utilising the coefficient of variation in acoustic pressure, this discrepancy is mitigated, and the data could be compared. Overall, the coefficient of variation in the 1.2 MHz field was found to be 29.9.%, largely owing to the peak rather than the oscillations 42.9% for the 666 kHz field. Further, higher frequency was quantifiably more consistently across the vertical axis. Figure 6B which depicts the profile of the field at the level of the cell-lines in the well and the profiles were observed to be similar.

### SDT impedes glioma cell growth

**Figure 7:**
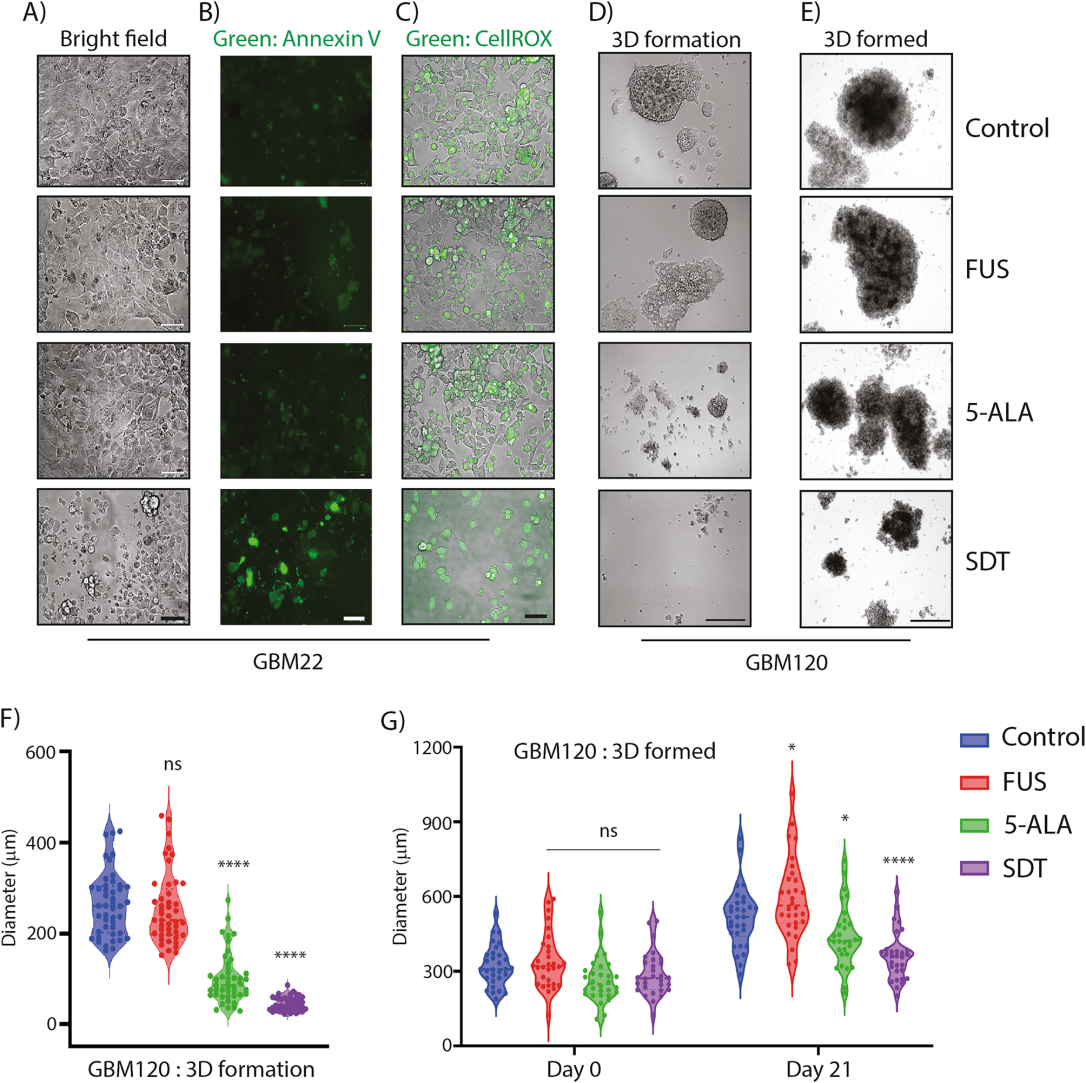
SDT induces cell death in primary patient derived glioma cells in vitro. (**A**) Representative bright field images of GBM22 cells 1.5 h post-treatment with/without 1 mM 5-ALA, with/without FUS (0.4 W/cm^2^, 30 s cumulative sonication, 10% DC, 90 ms pulse length), and both (SDT). Scale bar = 125 μm. (**B**) Representative fluorescent images only of GBM22 cells, pre-treated with Annexin V-FITC apoptosis marker, 1.5 h post-treatment with/without 1 mM 5-ALA, with/without FUS (0.4 W/cm^2^, 60 s cumulative sonication, 10% DC, 90 ms pulse length), and both (SDT). Scale bar = 125 μm. Also see Supplementary Fig. S6. (**C**) Representative overlaid bright field and fluorescent images of GBM22 cells, pre-treated with CellROX dye, 1.5 h post-treatment with/without 1 mM 5-ALA, with/without FUS (0.4 W/cm^2^, 30 s cumulative sonication, 10% DC, 90 ms pulse length), and both (SDT). Scale bar = 125 μm. (**D**) Representative bright field images of GBM120 3D neurospheres. The dissociated GBM120 cells were pre-treated with indicated conditions as in A and allowed to form neurospheres over 21 days. n = 3 biological replicates. Scale bar = 200 μm. (**E**) Representative bright field images of GBM120 3D neurospheres. The indicated treatments as in A were carried out on pre-formed 3D neurospheres and allowed to grow over a further 21 days. n = 3 biological replicates. Scale bar = 200 μm. (**F**) Quantification of D across all n = 3 replicates. The diameter of the neurospheres were quantified using ImageJ. The significance of the differences was measured using one-way ANOVA with Dunnett’s multiple comparisons. ****p < 0.0001; ns: not significant. (**G**) Quantification of the diameter of formed neurospheres on Day 0 and Day 21 across all n = 3 replicates. The diameter of the neurospheres were quantified using ImageJ. The significance of the differences was measured using two-way ANOVA with Bonferroni’s multiple comparisons. ****p < 0.0001; *p < 0.05; ns: not significant.

A previous study has utilised U87MG and U251 cells to establish SDT parameters^31^. However, the validity of both U87MG and U251 cells as glioma models have come under serious scrutiny^32, 33^. Hence, to establish the effect of SDT on state-of-the-art glioma cells, primary patient derived cells GBM22 and glioma stem neurospheres GBM120 were utilised. Upon SDT exposure, GBM22 cells exhibited marked cell death (Fig. 7A) along with enhanced Annexin V apoptotic signal (Fig. 7B & Supplementary Fig. S6). The cell death phenotype was not observed upon individual exposure to photosensitiser 5-ALA or focused ultrasound alone suggesting the combination of 5-ALA and FUS is required to induce apoptosis. Interestingly, apoptotic signal was exclusively present in the SDT samples with proportional increase observed with increasing dose of FUS (Supplementary Fig. S6). Furthermore, SDT exposure led to increased reactive oxygen species induction as evidenced by positive signal with CellROX (Fig. 7C). SDT induced ROS upregulation in 100% of attached live GBM22 cells (Fig. 7C). To understand if SDT exposure can target 3D glioma stem neurospheres, GBM120 cells were treated with or without 1 mM 5-ALA, FUS, and SDT. The cells were then cultured over 21 days and allowed to form 3D neurospheres in stem media. Interestingly, the SDT exposed population of GBM120 cells exhibited the smallest 3D spheroids compared to the 3 controls. 1 mM 5-ALA alone did exhibit some toxicity to GBM120 cells, but SDT treatment was the most effective in reducing glioma stem neurosphere growth (Fig. 7D,F). Similar results were obtained when GBM120 pre-formed 3D neurospheres were exposed to SDT (Fig. 7E,G). 5-ALA alone toxicity was modest but significant in the 21-day cohort and SDT treatment remarkably affected 3D spheroid growth progression over 21 days. SDT-treated cohort did show a very modest yet statistically significant growth over 21 days suggesting incomplete cell kill (Fig. 7G). Cells lysed 1.5 h post treatment showed an increase in phospho-ERK signal in 5-ALA, FUS, and SDT treatment groups compared to control while a very modest increase in phospho-AKT Ser473 and Thr308 were observed in 5-ALA treatment alone (Supplementary Fig. S7). Poly-D-lysine used to ensure immobilization of neurospheres for sonication altered the distribution of growth patterns from spheroids to more irregular patterns in some neurospheres (Fig. 7D,E). This prevented the accurate calculation of volume; hence diameter was used as a simplified measure and a greater sample size was collected to mitigate the effects of irregular growth. Since the centre of the well saw the highest peak pressure and acoustic intensity compared to well periphery (Fig. 3C), neurosphere sampling and imaging was conducted from the centre outwards, so that the results reflected the most consistent and predictable FUS dose parameters available within the current system. Neurosphere sampling was conducted via averaging 2 perpendicular diameters of the 5 neurospheres closest to the centre of each well, the strongest point of the FUS peak pressure map. A total of 45 neurospheres were measured per condition every 3 days for 21 days over 3 biological replicates with 3 technical replicates in each.

## Discussion

An understanding of the physical properties affecting accurate determination of FUS dose and consistency in dose application to cells is crucial to translatability and comparability of in vitro SDT experiments. Systems are often primarily designed to investigate one physical property (e.g. standing waves), as opposed to primarily designing the system for in vitro cell testing and then subsequently investigating the physical properties in the setup, as in ours. This creates difficulty in the incorporation of literature findings into in vitro testing setups for SDT. Further studies should be undertaken to refine similar experimental setups and tumour models for the purpose of rapid testing and optimisation of sonication parameters. With further understanding of the physical and cellular mechanisms involved, in vitro data may inform the evolution of ideal human trial FUS parameters, a significant factor in the clinical application of SDT.

Limited data exists on the impact of SDT in inducing cell apoptosis. For this reason, standard parameters for cell testing were derived from the most efficacious single-transducer pulsed-FUS parameters in rats^24^. A lower acoustic intensity was utilised to facilitate testing (Table 1), however, duty cycle, pulse length, and cumulative sonication dose were broadly maintained. The impact of both rat skulls and standard TC treated polystyrene plates will both act to reduce the target FUS intensity, though the degree may vary. It is important that in vitro experimental design and parameter selection is realistic when translated to patients. No thermal effect was seen at the selected intensity; alteration to duty cycle may have implications on skull heating effects when translated to human trials. There is an inverse relationship between duty cycle and treatment duration for a set cumulative sonication. We estimated that patients may not tolerate a treatment of over 90–100 min, for 18 points to be sonicated as in Wu et al.^25^, a 10% duty cycle was selected to apply 30 s of cumulative sonication to each point in this treatment duration.

**Table 1:** Comparison of focused ultrasound parameters (acoustic intensity, pulse length, duty cycle, and cumulative sonication time) used by Wu et al.^25^ to those included in this study.

Study	Acoustic Intensity (W/cm^2^)	Pulse length (ms)	Duty cycle (%)	Cumulative sonication (seconds)
This paper	0.4	90	10	30
Wu et al.^25^	5.5	86	8.6	24

The exact mechanism of PpIX activation and wider cellular impact of focused ultrasound is yet to be fully described. Further development of in vitro systems will allow the investigation of these mechanisms. This system may be used to examine the cellular effects of various combinations of focused ultrasound parameters in comparison to in vivo trials when standardised for the level of PpIX activation. The use of this surrogate marker for cavitation-induced sonoluminescence accounts for differences in ultrasound dynamics in vivo, for instance the impact of circulating oxygen. Though this system cannot currently examine the physical mechanism of sonodynamic therapy, it provides a strong basis for studies into the cellular mechanism and its efficacy.

The mitigation of standing waves is one of the key factors within FUS experimental setups. Standing waves create consistent nodes and anti-nodes which may have amplified effects; however, these are predictable and can be measured. Sampling cells from known nodes allows for estimation of pressures they have been exposed to in setups in which standing waves have not yet been fully eliminated. Further development of cell-safe methods of establishing a full fluid column will improve the concordance of field mapping simulations to the experience of cells. The use of PVA gels to eliminate or displace the fluid-air boundary in a sealed container is currently the most developed option, with the use of ultrasound absorbent material to prevent dose reflection also under investigation. This is discussed further in the supporting manuscript.

The needle hydrophone used to generate field maps required a minimum of 400 μL within each well, to prevent damage to the equipment. Most cells tests are undertaken with 100 μL of media in each well, which is expected to cause further standing wave reflection in simulations. The X and Z axis movements of the NH may have also disrupted the water’s surface, causing scattering of returning waves in field mapping results. To reduce this, greater intervals between NH detection following movement were introduced. The comparison of more complex setups to simulations may improve dose estimations where measurement via a NH is not possible, for instance sealed and submerged cell enclosures.

5-ALA treatment activated the proliferative AKT and ERK signalling (Supplementary Fig. S7). This was expected as 5-ALA is an amino acid derivative and potentially a nutrient. The reduced growth phenotype in 5-ALA alone cohort may be due to toxic metabolites accumulation over time in the cell media (Fig. 7D–G). Since GBM120 primary neurospheres are partly suspension cultures, we were unable to replace the media to remove the excess 5-ALA. This toxicity may not be an issue in patients as the exogenous amino acid is excreted out quickly and is FDA-approved for ingestion. Furthermore, SDT cohort had a significantly reduced growth profile than 5-ALA cohort alone in both formed and formation assays (Fig. 7D,F,H). Ultrasound cannot be focussed on free suspension spheroids as the spheroids bounce away from the focal field of the ultrasound. Therefore, immobilisation of 3D spheroids was crucial for our sonication studies. Poly-D-lysine provides a very gentle semi-suspension condition for the 3D spheroids and does not allow for media changes or fresh media additions without significantly affecting the integrity of the 3D spheroids. Interestingly, only phosphorylated ERK was observed upon FUS treatment (Supplementary Fig. S7) which could be a response to the stress. While FUS alone stress is potentially non-fatal, the combination of 5-ALA and FUS is indeed highly toxic and tips the scales for glioma (Fig. 7). This apoptotic cell death observed in SDT alone could be partly due to excess ROS production. The slight increase in FUS–alone cohort over 21 days (Fig. 7G) is probably due to a broader distribution of initial diameter of neurospheres. The SDT treated cohort also exhibited a modest growth over 21 days suggesting that some cells remain proliferative within the neurospheres. It would be interesting to check if combination with chemotherapy could alleviate this modest growth.

An understanding of factors influencing ultrasound fields and the mitigation of interference is necessary for the accurate determination of cell dosing in in vitro studies and the translatability to clinical sonodynamic therapy. Herein, we report an optimised system that can induce death in glioma 2D and 3D glioma cells in vitro and sets the basis for further understanding of SDT and its therapeutic potentials in cancer.

### Supplementary Information

Supplementary Information.

## Data Availability

All data that support these findings of the present study are included in this manuscript and its supplementary information files. Further information is available upon request to the corresponding author, James Joseph.
